# Alimentação e sustentabilidade em perspectiva multidimensional: percepções e práticas de jovens rurais

**DOI:** 10.1590/0102-311XPT101623

**Published:** 2025-06-09

**Authors:** Paula Bernardes Machado, Carolina Martins dos Santos Chagas, Amábela de Avelar Cordeiro, Elisa Guaraná de Castro, Thais Salema Nogueira de Souza

**Affiliations:** 1 Universidade Federal de Lavras, Lavras, Brasil.; 2 Universidade Federal do Rio de Janeiro, Macaé, Brasil.; 3 Universidade Federal Rural do Rio de Janeiro, Seropédica, Brasil.; 4 Universidade Federal do Estado do Rio de Janeiro, Rio de Janeiro, Brasil.

**Keywords:** População Rural, Juventude, Sistema Alimentar Sustentável, Rural Population, Youth, Sustainable Food System, Población Rural, Jovem, Sistema Alimentario Sostenible

## Abstract

A reconfiguração de sistemas alimentares com enfoque na sustentabilidade multidimensional é fundamental para a garantia da alimentação adequada e para a saúde do planeta, sendo as juventudes rurais os atores estratégicos para essas mudanças. O presente trabalho teve como objetivo compreender as percepções, as práticas e os elementos de mobilização de jovens rurais em relação à alimentação e à sustentabilidade. Trata-se de uma pesquisa qualitativa e parte integrante do projeto *Educação Alimentar e Nutricional com Juventudes: Mobilização, Redes e Cooperação Institucional*, no qual foram realizadas entrevistas semiestruturadas individuais com jovens do meio rural, examinadas por meio da Análise de Conteúdo. A partir do *corpus*, emergiram quatro categorias: (i) Produção e comercialização de alimentos: entre a subsistência e as relações afetivas; (ii) Relação saudável com a terra e todas as formas de vida; (iii) Coletividade, justiça e solidariedade; e (iv) Alimentação e sustentabilidade: propostas de mobilização. As pessoas entrevistadas demonstraram preocupação com todas as etapas do sistema alimentar, e suas percepções e práticas foram pautadas pela sustentabilidade nas dimensões ambiental, econômica, social e cultural. Reflexões convergentes, complementares e significativas - geradas por meio deste estudo - inspiraram um ensaio para a ressignificação do conceito de alimentação sustentável, que certamente poderá contribuir para a mobilização de outros jovens e aprofundamentos sobre a temática.

## Abstract (en)

The reconfiguration of food systems with an emphasis on multidimensional sustainability is fundamental to ensuring adequate nutrition and the health of the planet, with rural youth serving as strategic agents for these changes. This study aimed to understand the perceptions, practices, and mobilization elements of rural youth regarding food and sustainability. It is a qualitative investigation that forms part of the project *Food and Nutritional Education with Youth: Mobilization, Networks, and Institutional Cooperation*, in which individual semi-structured interviews were conducted with rural youth and analyzed using Content Analysis. Four categories emerged from the data: (i) Food production and marketing: between subsistence and affective relationships; (ii) A healthy relationship with the land and all forms of life; (iii) Collectivity, justice, and solidarity; and (iv) Food and sustainability: mobilization proposals. The interviewees expressed concern about all stages of the food system, and their perceptions and practices were guided by sustainability in its environmental, economic, social, and cultural dimensions. The convergent, complementary, and significant reflections generated by this study inspired an essay to reframe the concept of sustainable food, which will undoubtedly contribute to the mobilization of other young people and further explorations of the topic.

## Resumen

La reconfiguración de los sistemas alimentarios desde un enfoque en la sostenibilidad multidimensional es fundamental para garantizar una alimentación adecuada y la salud del planeta, y la juventud rural son los agentes estratégicos para estos cambios. Este estudio tuvo como objetivo identificar las percepciones, las prácticas y los elementos de movilización de la juventud rural en relación con la alimentación y la sostenibilidad. Se trata de una investigación cualitativa, que conforma el proyecto *Educación Alimentaria y Nutricional con Jóvenes: Movilización, Redes y Cooperación Institucional*, en el que se realizaron entrevistas individuales semiestructuradas con la juventud rural y la aplicación de análisis de contenido. Del corpus se obtuvieron cuatro categorías: (i) Producción y comercialización de alimentos: entre relaciones de subsistencia y afectivas; (ii) Relación sana con la tierra y todos los modos de vida; (iii) Colectividad, justicia y solidaridad; y (iv) Alimentación y sostenibilidad: propuestas de movilización. Los entrevistados mostraron preocupación por todas las etapas del sistema alimentario y sus percepciones y prácticas se guiaron por la sostenibilidad en las dimensiones ambiental, económica, social y cultural. Las reflexiones convergentes, complementarias y significativas generadas en este estudio inspiraron un ensayo para la redefinición del concepto de alimentación sostenible, que, sin duda, puede contribuir a la movilización de otros jóvenes y a la profundización en el tema.

## Introdução

A reformulação dos sistemas alimentares representa um desafio global, exigindo estratégias de enfrentamento estruturais e sistêmicas ^1^. A intensa produção do agronegócio avança e revela lacunas em relação à soberania e segurança alimentar e nutricional das nações. Isto é, não garante a realização plena do direito humano à alimentação adequada (DHAA) e o direito dos povos em decidir e preservar suas estratégias de produção, distribuição, consumo e práticas alimentares tradicionais de base sustentável ^2^ ^,^ ^3^.

O sistema agroalimentar hegemônico é determinante para emissão dos gases de efeito estufa, mudanças climáticas globais, desmatamento, destruição da biodiversidade e uso irresponsável de água potável; bem como para a reprodução e ampliação de desigualdades sociais, impedindo o acesso à terra e à água, e aprofundando formas precárias e desiguais de renda - ou mesmo para a exploração não remunerada, promovendo pobreza e fome, em especial para mulheres e crianças ^4^ ^,^ ^5^. Também está fortemente associado à obesidade, desnutrição e insegurança alimentar e nutricional ^6^. 

Urge reconfigurar esses sistemas para garantir alimentação adequada a todas as pessoas e contribuir para a saúde do planeta a partir de uma perspectiva de sustentabilidade que considere suas dimensões ambiental, social, cultural e econômica ^2^ ^,^ ^3^. 

Vários países têm buscado caminhos para alcançar uma alimentação saudável e sustentável, inclusive o Brasil ^7^ ^,^ ^8^. Nesse campo, são encontrados diferentes conceitos que relacionam alimentação e sustentabilidade: Dietas Sustentáveis, Dietas Saudáveis e Sustentáveis, Dietas Saudáveis a partir de Sistemas Alimentares Sustentáveis. Estes incluem orientações complementares ou até mesmo divergentes, abordando ou não a relação com sistema alimentar, foco na dimensão biológica da alimentação, menção a grupos alimentares e maior importância à dimensão do meio ambiente ^1^ ^,^ ^9^ ^,^ ^10^ ^,^ ^11^ ^,^ ^12^ ^,^ ^13^ ^,^ ^14^ ^,^ ^15^. A diferenciação conceitual chama atenção para um plano de disputa nos campos político e acadêmico, revelando a ausência de consenso.

Os debates acerca da sustentabilidade contam com contribuições das juventudes que têm se inserido em espaços políticos relacionados a essa pauta ^16^. Destaca-se o papel de jovens que vivem no meio rural, que compartilham das lutas pelo direito à terra, à água, à soberania e segurança alimentar e nutricional; são parte determinante na transição agroecológica, no fortalecimento da agricultura familiar ^17^ e são atores fundamentais na agenda da sustentabilidade. A alimentação também é presente nos diálogos entre jovens rurais em espaços políticos e, por vezes, está relacionada à dimensão da sustentabilidade ^18^ ^,^ ^19^ ^,^ ^20^ ^,^ ^21^ ^,^ ^22^.

Diante disso, cabe questionar: será que as juventudes do campo no Brasil, que produzem e comercializam alimentos, poderiam agregar novos elementos para a tessitura do conceito de alimentação sustentável pautado em suas percepções e práticas? Quais dimensões poderiam emergir dos processos de escuta? Quais elementos podem engajar outros jovens do meio rural na temática?

Acredita-se na potencialidade desta pesquisa ao promover espaços de discussões sobre alimentação e sustentabilidade a partir das reflexões de atores sociais e políticos dos sistemas alimentares - neste caso, as juventudes rurais. Este trabalho tem como objetivo compreender as percepções, as práticas e os elementos de mobilização de jovens rurais em relação à alimentação e à sustentabilidade.

## Metodologia

Este estudo integra o projeto *Educação Alimentar e Nutricional com Juventudes: Mobilização, Redes e Cooperação Institucional*, comumente conhecido como *Movimento Comer Pra Quê?*, que foi aprovado pelo Comitê de Ética em Pesquisa (CAAE: 36202620.1.1001.5148) e financiado pelo Ministério da Cidadania. As ações do projeto são orientadas pelos pressupostos pedagógicos da educação Freiriana, em especial a dialogicidade, a problematização e a construção coletiva de conhecimentos ^23^.

Esta é uma pesquisa qualitativa cujo critério de inclusão integrou jovens entre 18 e 29 anos das cinco regiões do país, residentes no meio rural e envolvidos com a produção ou comercialização de alimentos. Entre os meses de agosto e outubro de 2021, realizou-se o contato com 57 organizações que desenvolvem atividades com jovens do meio rural. Nessa fase, foi enviado um formulário online com informações sobre a pesquisa e solicitação de indicação de jovens para participarem do processo, caracterizando uma amostra de conveniência. Adotou-se a técnica de amostragem “bola de neve”, utilizada para facilitar o acesso a grupos difíceis ^24^, possibilitando o mapeamento de 53 jovens rurais. As mulheres demonstraram maior interesse em participar das atividades (n = 44), e esse resultado pode se relacionar com a obstinação feminina frente à masculinização do campo - um dos fatores que estimula o êxodo rural de mulheres jovens a partir dos 20 anos em função dos estereótipos e papéis de gênero atribuídos ao trabalho do campo ^25^.

Em novembro e dezembro de 2021, foram realizadas entrevistas semiestruturadas individuais ^26^ com duração média de 50 minutos, em ambiente on-line, junto aos dez jovens que aceitaram participar da pesquisa via Termo de Consentimento Livre e Esclarecido (TCLE). O repertório temático foi repetido com cada participante até o alcance da saturação. As entrevistas foram transcritas e analisadas manualmente por meio da Análise de Conteúdo ^27^. As unidades de significados e sentidos relativos ao tema foram selecionadas após a análise comparativa de cinco pesquisadoras.

## Resultados e discussão

Concordaram em participar da entrevista dez jovens, com idades entre 18 e 29 anos, sendo a maior parte com idade entre 21 e 25 anos (n = 7), mulheres (n = 9), não brancas (n = 7) e residentes na zona rural de diferentes regiões do país, conforme Quadro 1. 

**Quadro 1:** Perfil social dos participantes. Brasil, 2022.

JOVEM	FAIXA ETÁRIA (ANOS)	GÊNERO	RAÇA	ESTADO CIVIL	REGIÃO DO PAÍS EM QUE RESIDE	ATIVIDADE DESEMPENHADA	RENDA FAMILIAR	PARTICIPAÇÃO EM COLETIVOS
J1	21-25	Feminino	Branca	Solteira	Sul	Produção e comercialização	Independe	Não
J2	18-20	Feminino	Branca	Solteira	Sudeste	Produção de alimentos	Depende	Coletivo Feira Agroecológica de Lumiar
J3	26-29	Feminino	Preta	Solteira	Nordeste	Produção de alimentos	Depende	Não
J4	21-25	Feminino	Amarela	Solteira	Sudeste	Produção de alimentos	Depende	Movimento dos Pequenos Agricultores
J5	21-25	Feminino	Parda	Casada	Sudeste	Produção de alimentos	Depende	Não
J6	21-25	Feminino	Parda	Solteira	Sudeste	Produção de alimentos	Depende	Não
J7	26-29	Feminino	Preta	Solteira	Sudeste	Produção e comercialização	Independe	Não
J8	21-25	Feminino	Parda	Solteira	Norte	Produção e comercialização	Depende	Movimento dos Pequenos Agricultores
J9	21-25	Masculino	Parda	Solteiro	Nordeste	Produção e comercialização	Depende	Não
J10	21-25	Feminino	Branca	Solteira	Centro-oeste	Produção	Independe	Projeto Agricultura na Cidade

A maioria estava envolvida com a produção (n = 7) ou com a produção e comercialização de alimentos (n = 3), e grande parte não participava de instituições ou coletivos (n = 6). 

A partir da sistematização do material e da análise do corpus, emergiram quatro categorias: I − Produção e comercialização de alimentos - entre a subsistência e as relações afetivas; II − Relação saudável com a terra e todas as formas de vida; III − Coletividade, justiça e solidariedade; e IV − Alimentação e sustentabilidade - propostas de mobilização.

### Produção e comercialização de alimentos: entre a subsistência e as relações afetivas

Para os jovens, “*a principal vantagem* [de plantar], *é o consumo sustentável principalmente, e também uma forma de você ajudar na renda* (...) *É uma forma de auxiliar nos gastos e tal*” (J9). A maior motivação dos entrevistados em produzir alimentos foi a própria subsistência e a de sua família: “[plantamos] *primeiro pela necessidade mesmo, de a gente produzir nosso alimento, os preços estão altíssimos*” (J4). Plantar seu alimento, tendo em vista os preços, se mostrou urgente. Produzir em variedade possibilitou autonomia alimentar aos jovens e sua família, mesmo que parcial, dado que eles se tornam cada vez mais independentes do supermercado: “*A gente vai para a roça, a gente planta mandioca, tem cacau, banana, essas coisas. É bom para o nosso consumo, porque as coisas estão um pouco caras, né?* (...) *para a gente poder consumir essas coisas, não comprar, então tem que plantar mesmo*” (J5).

A agricultura, principalmente a de pequena escala, é fundamental para a população rural, pois contribui para o acesso aos alimentos. Tendo em vista que essa população é mais acometida pela insegurança alimentar e nutricional, a produção para o autoconsumo, geralmente caracterizada pela diversificação, é fundamental para a garantia da segurança alimentar e nutricional das famílias no meio rural ^28^ ^,^ ^29^ ^,^ ^30^.

Os entrevistados envolvidos com a comercialização de alimentos consideraram uma importante fonte de renda, apesar de incerta e insuficiente: “*...a gente produz... porque camponês é teimoso e planta mesmo, toda vez vai plantar e corre o risco de perder, mas planta, né? Mas não sabe quando que vai vender e como que vai vender, né? E o ideal seria a gente ter a condição de plantar sem ter prejuízo*” (J8), “*...acho que a dificuldade é meio que essa, conseguir dar conta de tudo a ponto de ser rentável*” (J1). Na agricultura familiar e camponesa, é comum o revezamento entre consumo e comercialização do que se planta de acordo com diferentes aspectos, sendo um deles a disponibilidade de mercados viáveis e os preços que são pagos por eles. Assim, os agricultores fazem diferentes escolhas, a depender das dificuldades, buscando estabilidade econômica ^31^. Além disso, a diversificação na produção, e consequentemente nas atividades, é um elemento necessário para que a sustentabilidade econômica se desenvolva, sendo presente nas práticas dos entrevistados ^32^.

Em um modelo de produção e comercialização ideal para os jovens, não haveria prejuízos financeiros, e o consumidor seria consciente dos desafios enfrentados pelos produtores, de forma a se aproximar e facilitar o processo e a emancipação do produtor: “[O modelo de produção e comercialização] *ideal com certeza* (...) *é um grupo de consumidores conscientemente responsáveis, por comprar uma cesta de alimento que eu decida o que vai entra*r (...) *Melhor ainda seria se essas pessoas aparecessem na minha propriedade para pegar as coisas*” (J1).

Este formato idealizado pelos participantes traz elementos das chamadas redes agroalimentares “alternativas” ou cidadãs, que segundo Rover & Darolt ^33^ (p. 21) englobam “*cooperação social e parcerias entre produtores e consumidores, encurtando as cadeias com um número reduzido (ou inexistente) de intermediários e uma limitada distância geográfica entre ambos*” e são consideradas inovações sociais que apontam caminhos para superação de barreiras impostas pelo sistema agroalimentar vigente. Destaca-se que a aproximação entre produtor e consumidor é um dos eixos da segurança alimentar e nutricional ^3^.

Apesar dos desafios, demonstrou-se amor pela produção de alimentos, respeito aos saberes ancestrais e valorização das práticas populares na agricultura vivenciadas em uma simples conversa com os vizinhos, pais ou avós, considerados mais importantes que as referências da universidade: “*Quando eu chego na faculdade para fazer Ciências Agrícolas* (...) *eu falo: caraca, essa galera não sabe nada, meu pai sabe mais que essa galera, minha avó sabia muito mais que o pessoal todo aqui* (...) *Eu já tenho as minhas referências, eu não quero isso* (...)*, as minhas referências são muito maneiras, de onde eu vim é muito legal*” (J7). Essa troca impulsionou os jovens a seguirem produzindo alimentos na lógica da Soberania Alimentar, de modo que, reproduzindo esses saberes, seguem preservando estratégias de produção, distribuição, consumo e práticas alimentares tradicionais ^2^: “*Tem uns senhorzinhos que moram aqui em volta, que super ajudam* (...) *Então a gente descobriu várias plantas novas para fazer chá, para tempero.* (...) *eram coisas que já estavam aqui, não foi nem coisa que a gente trouxe* (...) *é planta que nasce espontânea.* (...) *ver todas essas plantas, esses potenciais, aqui no quintal e eu nem percebia, eu acho que isso foi o mais legal. E a troca com as outras pessoas aqui do bairro também, que aí eles nos ensinam, falam: essa mandioca é tal coisa, e aí você vai aprendendo várias coisas sobre mandioca, você fala: o que é isso? Eu só fui pedir um negócio ali para o vizinho, e aí você volta com uma aula de mandioca*” (J6). 

Na contramão do modelo dominante, a resistência e valorização dos saberes e fazeres tradicionais e populares possibilita um resgate cultural e histórico nos territórios. Por meio dessa resistência os jovens produzem alimentos com respeito aos bens naturais e socioculturais, peça substancial para a sustentabilidade na agricultura “não convencional” ^34^. 

### Relação saudável com a terra e todas as formas de vida

Considerou-se que a alimentação, para ser sustentável, deve ser oriunda de um sistema que preserve e promova não só a saúde humana, mas também a da terra e a de todas as outras formas de vida do planeta. Consequentemente, isto envolve uma produção “*com sementes crioulas, com respeito, em comunhão com a terra, com o solo, com o ar, com a água, com o clima, com a fauna e com a flora local...*” (J2) e sem uso de “veneno”, como comumente se referem aos agrotóxicos. Para os participantes, não há um único modelo de produção e comercialização que se desenvolva dessa maneira, considerada ideal; no entanto, desaprovam o agronegócio e a prática do monocultivo: “*...eu tenho para mim que a sustentabilidade é o que é a agroecologia, mas acho que existem vários nomes e a gente não precisa se ater a um nome específico. Mas é isso, terra sadia, planta sadia, pessoa sadia. Então, se esse alimento é produzido com veneno, se esse alimento é produzido num sistema de monocultivo,* (...) *se ele não tem todos os nutrientes ali depositados, ele não vai ser um alimento tão rico como a gente poderia ter. E eu acho que é isso, se ele gera resíduos também, uma coisa a se ver, pode ser um alimento super orgânico, maravilhoso, sei lá o que, mas se ele é embalado em plástico e depois não vai ter o que fazer*” (J1).

De fato, o sistema baseado no agronegócio gera externalidades negativas para o meio ambiente e para a saúde ^6^ ^,^ ^35^. Os impactos ambientais são reconhecidos a nível global, como a emissão de gases de efeito estufa, a devastação da biodiversidade, o desmatamento e o desperdício de água potável. Além disso, é sabido que toda a população mundial está exposta aos agrotóxicos, seja de forma aguda no trabalho agrícola ou pela contaminação ambiental, da água, do ar e dos alimentos, o que tem sido associado a distúrbios em sistemas fisiológicos e ao aumento de doenças crônicas não transmissíveis (DCNT) ^36^ ^,^ ^37^ ^,^ ^38^. 

Por outro lado, sistemas baseados na agricultura orgânica, assim como nas demais sob a denominação de biológica, ecológica, biodinâmica, agroecológica e natural, são comprometidos a não usar agrotóxicos e se apresentam mais ecológicos, rentáveis e com mais benefícios sociais em relação ao sistema hegemônico, sendo assim mais sustentáveis em diferentes dimensões: “*Não são alimentos que se utiliza agrotóxico* (...) *não tem a utilização de produtos que venham causar danos nem ao solo, nem à terra aqui, nem a gente que produz, nem à própria pessoa que vai consumir, porque a gente não usa nenhum tipo de agrotóxico*” (J3). Os alimentos produzidos nesses sistemas contribuem para a saúde humana e diminuem o risco de DCNT, pois apresentam maior quantidade de compostos bioativos e menor, ou nenhuma, quantidade de produtos tóxicos ^39^ ^,^ ^40^.

Nessa perspectiva, a partir do “*...contato tanto com o agronegócio, quanto com a produção agroecológica, quanto com a produção agroflorestal,* (...) *a produção ideal mesmo é quando a gente consegue respeitar todos os modelos de vida, quando a gente consegue produzir alimento para nós, seres humanos, sem agredir esse planeta, porque eu acredito que somos um só, não existe essa individualidade de ser humano quando o assunto é a terra*” (J2). 

Para além da produção de alimentos de forma sustentável, a geração de lixo, especialmente por meio de embalagens, foi relevante para os entrevistados, que buscavam utilizar ou mesmo desenvolver embalagens respeitosas ao meio ambiente: “*Um desafio foi pensar nas embalagens* (...) *eu comecei a fazer as embalagens de fibra de banana. Então tem dado muito certo, eu estou conseguindo embalar praticamente todos os produtos com fibra de banana* (...) *eu reaproveito a fibra externa, boto para secar, faço o processo de secagem natural mesmo, boto num varal e sol, ou sombra, e aí eu consigo embalar os produtos todos, então eu consegui diminuir custo, consegui não usar plástico nenhum*” (J7).

O descarte excessivo, o desperdício e o acúmulo de lixo resultantes do modelo hegemônico de produção e consumo distanciam a sociedade da sustentabilidade ^41^. Embalagens descartáveis usadas em bebidas, carnes, frutas e legumes acarretam bilhões de toneladas de lixo e sérios danos ao meio ambiente, cujas estratégias de enfrentamento envolvem práticas legislativas de proibição do uso de plásticos, conscientização dos consumidores e pesquisas para o desenvolvimento de materiais substitutos ^42^. Em circuitos de comercialização baseados na agroecologia e em outras redes agroalimentares chamadas “alternativas”, prioriza-se a redução do uso de embalagens plásticas, a venda de alimentos a granel e o uso de embalagens ecológicas ou retornáveis ^33^. 

As perspectivas e diversas práticas adotadas pelos jovens se ancoram na dimensão ambiental da sustentabilidade no sentido de conservar o meio ambiente, a biodiversidade, suprimir a poluição ^43^ e, ainda dialogam com alguns eixos da segurança alimentar e nutricional, sendo: (i) produção sustentável de alimentos; (ii) preservação dos recursos naturais; e (iii) respeito à sociobiodiversidade ^3^.

### Coletividade, justiça e solidariedade

Desenvolver as atividades de produção e comercialização de alimentos a partir de um grupo de pessoas foi considerado o ideal pelos jovens e os motiva a seguir com as práticas, vislumbrando a possibilidade de agregar mais sujeitos a essa coletividade: “*E nessa parte das ações serem bem mais práticas* (...) *Das ações entre vizinhos, dessa criação de redes, da criação da rede ecológica, de viver isso na prática* (...) *E poder estar abrindo um caminho... para mim está sendo difícil, mas talvez para quem venha depois não seja tão difícil assim. Sejam amigos que venham integrar essa coletividade, sejam os próprios filhos* (...) *Logo desejo que a gente consiga se manter tão bem que a gente consiga chamar outras pessoas*” (J1).

Os resultados convergem com fundamentos da sustentabilidade social tendo em vista que se considerou o direito de todos os cidadãos a usufruir dos bens para que tenham uma vida digna, nesse caso: “[O modelo de produção ideal é] *uma produção coletiva, agroecológica, sem exploração* (...) *partindo do princípio de que a terra é de todos e a gente pode sim produzir pensando nessa questão da alimentação e não do lucro. No agronegócio a gente vê muito as pessoas trabalhando muito e com uma qualidade de vida péssima*” (J4) ^44^ ^,^ ^45^. Ressalta-se que a organização coletiva catalisa práticas agrícolas ecológicas e ainda favorece a superação dos desafios para acessar os mercados. Ou seja, a coletividade mobiliza o aprofundamento da sustentabilidade econômica, social e ambiental ^46^ ^,^ ^47^.

O trabalho justo foi considerado ponto central para que um sistema de produção e comercialização de alimentos seja sustentável. Um produtor que não é explorado, um processo de trabalho que se desenvolve “*sem patrão*” (J1) em detrimento a um modelo em que a carga de trabalho exaustiva afeta a qualidade de vida do trabalhador, emerge como contraponto entre um sistema que objetiva produzir comida para todos e outro que visa apenas lucrar: “*...*[a produção de alimentos] *é totalmente feita por nós, então feita por trabalhadores felizes, ela não explora ninguém a não ser a minha própria mão de obra, e do meu marido, e a gente gosta do que a gente faz*” (J1).

De fato, estabelecimentos rurais que produzem na lógica do agronegócio mantêm um padrão histórico de subordinação na relação empregador e empregado, que tem caminhado na supressão dos direitos trabalhistas com o avanço neoliberal ^48^ ^,^ ^49^. Por outro lado, a agricultura familiar, camponesa e tantos outros trabalhadores do campo, resistem na trajetória do seu processo autônomo de geração de excedentes e renda ^50^. A lógica de trabalho priorizada pelos jovens se comunica com dimensões socioculturais, fundamentais para a sustentabilidade do sistema, quais sejam a geração de trabalho, distribuição igualitária de renda e qualidade de vida ^32^ ^,^ ^44^ ^,^ ^51^. 

De forma mais aprofundada, um sistema de produção e comercialização de alimentos vislumbrado pelos jovens deveria ser organizado por meio de cooperação e solidariedade. Nesse sistema, a ajuda mútua, as trocas solidárias entre vizinhos e até mesmo com o consumidor permitiriam o seu fortalecimento sustentável. No entrelace entre solidariedade e sustentabilidade, alguns elementos foram compreendidos neste arranjo: (i) proximidade ao consumidor; (ii) alimentos acessíveis, especialmente aos indivíduos socioeconomicamente vulneráveis - e não um nicho de mercado; (iii) não envolvimento de atravessadores; e (iv) ações coletivas de doação de alimentos. Reconhece-se que “*...para vender para o mercado, para o mercado vender, a gente que tem o produto agroecológico, orgânico, o mercado vende e não chega para a população que precisa. Só quem tem condição consegue alcançar um alimento de qualidade hoje nos grandes mercados. Então, a gente tem essa política do produtor direto com o consumidor, sem terceirização, sem terceiros. E aí a gente vende assim. Aí agora, no período da pandemia, a gente tem tido projetos tanto do MPA* [Movimento dos Pequenos Agricultores], *quanto do MST* [Movimento dos Trabalhadores Rurais sem Terra], *para fornecer cestas para as pessoas com necessidade* (...) *às vezes a gente faz doação mesmo*” (J8). A solidariedade foi um compromisso presente nas entrevistas e se manifestou em dois princípios éticos considerados essenciais para a dimensão social da sustentabilidade: a solidariedade sincrônica, com a geração atual, refletida em atos de doação de alimentos, e a solidariedade diacrônica, que se traduziu no desejo de integrar a geração futura em uma coletividade, a fim de facilitar o seu trabalho e qualidade de vida ^45^ ^,^ ^51^.

### Alimentação e sustentabilidade: propostas de mobilização

Para mobilizar outros jovens rurais em relação a alimentação e sustentabilidade, os entrevistados apontaram atividades presenciais e virtuais: “*acho que precisa de ações presenciais no interior* (...) *a presença é bem importante* (...) *não sei se a população daqui se interessaria por uma semana de debates, mas talvez se interessaria, daqui a pouco a gente chama uma feira, que é uma feira da biodiversidade, que também tem música* (...) *fazer eventos que sejam focados nisso, mas que eles não sejam chatos, sabe? Que seja uma coisa bem legal, que tenha movimento, som, coisas divertidas acontecendo, feira, comida. Uma festa, assim, quase, para chamar gente. Eu aposto nisso*” (J1); “*Hoje pelo meio virtual é muito mais fácil de a gente conseguir atrair esses jovens. Até porque eu vejo isso aqui: com essa questão desse mundo virtual que a gente está vivendo* (...) *os jovens, eles já têm uma certa facilidade. Então a gente precisa encontrar o meio adequado de como atrair*” (J3). As redes digitais podem se configurar como aliadas nesse contexto visto que a apropriação do ciberespaço tem demonstrado potencial para mobilização social ^52^.

Independente da modalidade, essas atividades devem ser dinâmicas, pautadas na realidade concreta - “*procurando o interesse que as pessoas têm*” (J10) - e no diálogo, considerando a inclusão de atividades artísticas e culturais: “*...a juventude gosta de coisa dinâmica, que chame atenção* (...). *A gente que trabalha com a juventude, sabe que a juventude não vai sentar e ficar escutando três horas de palestra*” (J8); “*Eu acho que dessa forma* (...) *procurando o interesse que as pessoas têm e ir correlacionando as coisas* (...) *em formato de conversa, aberta, que a gente consiga entender, conversar com todo mundo, trocar as experiências*” (J10).

As sugestões se relacionam com fundamentos da educação alimentar e nutricional e da educação popular. Ações baseadas no diálogo e contextualizadas à realidade dos sujeitos exigem relações horizontais e contribuem para o desenvolvimento do senso crítico e de estratégias para lidar com diferentes situações cotidianas, tecendo um paralelo entre reflexão e ação. Atividades dinâmicas pressupõem o envolvimento dos sujeitos e a aprendizagem criativa, de modo que, participando ativamente dos processos, aumentam sua capacidade de transformação da realidade ^23^ ^,^ ^53^. De forma convergente, a arte pode ser uma ferramenta promotora do diálogo horizontal, indicada como facilitadora da compreensão e incentivadora de reflexões, com capacidade de produzir e difundir conhecimentos ^54^ ^,^ ^55^.

A temática mais emergente diz respeito à valorização da vida no campo, tendo em vista maior aproximação com o alimento: “*...eu acho que é muito melhor ficar no campo produzindo a própria comida do que ficar nessas grandes cidades, num sistema de cao*s (...) *O campo traz essa proximidade do alimento. É algo de grande valor. Então conseguir apresentar isso para esses jovens que são herdeiros desse modelo de produção, acho que seria muito importante para perpetuar essa prática*” (J2). Alguns autores evidenciam uma valorização cultural, social e de trabalho dos meios de vida urbano em detrimento ao rural. No entanto, os jovens rurais elencam diversas vantagens em viver neste meio comparados à vida urbana - entre eles a alimentação diversificada e as relações de troca entre as pessoas ^56^ ^,^ ^57^ ^,^ ^58^. Foi enfatizada pelos jovens deste estudo a necessidade de valorizar o campo como um lugar favorável para se viver.

Os desafios encontrados na prática da produção e comercialização de alimentos também foi pauta importante para os jovens, no sentido de promoção da sua emancipação financeira ^17^ ^,^ ^59^: “*Eu entendo que a produção e a comercialização, tanto porque a juventude do campo tem uma dificuldade na produção de renda para ela, que é uma coisa mais familiar e tal. E eu acho que entender o processo da produção e da comercialização dos alimentos seria uma coisa muito importante e necessária para a juventude*” (J8). Essa independência tende a romper com conflitos familiares gerados no meio rural pela subordinação de jovens à estrutura patriarcal imposta, que os desmotiva a permanecer no campo produzindo alimentos. Quando há esse rompimento, o jovem passa a ocupar lugar de destaque na produção e a diversificar as atividades na propriedade para além da produção agrícola ^60^. 

As propostas de mobilização extraídas da análise estão em consonância com princípios e bases epistemológicas da educação alimentar e nutricional e da educação popular, tais como dialogicidade, conscientização, processos ativos e contextualizados à realidade concreta dos indivíduos e transformação da realidade, de forma que poderão contribuir para a autonomia de jovens rurais no campo temático proposto ^23^ ^,^ ^53^.

### Ressignificando conceitos

Nos resultados encontrados, identificaram-se aspectos em comum com os conceitos já existentes que relacionam alimentação e sustentabilidade, a saber: baixo impacto ambiental; garantia da segurança alimentar e nutricional; preocupação com as gerações futuras; respeito à biodiversidade; acessibilidade financeira; justiça; saúde; sustentabilidade ambiental e social; sistemas alimentares sustentáveis; e diversidade alimentar. 

Extrapolando esses conceitos, os jovens trouxeram pontos significativos que contribuem para o aprofundamento sobre a alimentação sustentável: acesso físico aos alimentos; sustentabilidade econômica com vistas à emancipação dos produtores rurais; consumidor consciente quanto à valorização dos produtos e à sazonalidade da alimentação; produtor consciente quanto aos impactos ambientais e socioculturais em suas práticas; valorização de saberes populares; preocupação com embalagens; produção em coletividade; amor em produzir alimentos.

Inspiradas nos achados, as autoras propõem um breve ensaio de ressignificação do conceito de alimentação sustentável e seus princípios orientadores, que poderão aprofundar o diálogo sobre a alimentação na perspectiva da sustentabilidade.

A alimentação sustentável é composta por uma diversidade de alimentos, em quantidade e qualidade suficientes, que são acessíveis física e economicamente; é derivada de um sistema alimentar que gera baixo impacto ambiental, em todas as suas etapas, e promove o uso responsável dos recursos naturais e humanos, a aproximação e conscientização do produtor e consumidor, a autonomia, a saúde das pessoas, o respeito à biodiversidade e à sazonalidade; alicerçada no direito humano à alimentação adequada e saudável e na segurança e soberania alimentar e nutricional, tendo por princípios:

(1) Emancipação dos sujeitos;

(2) Justiça econômica e social;

(3) Coletividade e solidariedade;

(4) Transformação da realidade para garantia da qualidade de vida da geração presente e futura;

(5) Valorização dos saberes e dos modos de vida no campo;

(6) Relevância das relações afetivas na produção de alimentos.

Determinados temas apareceram com menor frequência nas entrevistas e também podem contribuir para o diálogo sobre a alimentação sustentável: reforma agrária; fome; consumo de ultraprocessados; e consumo de carnes. No entrelaçamento conceitual, percebeu-se a ausência de eixos da soberania alimentar, que são fundamentais para a sustentabilidade de um sistema alimentar. Por outro lado, a soberania alimentar esteve presente transversalmente em muitas das contribuições dos participantes.

## Considerações finais

Em suas percepções e práticas, os jovens demonstraram cuidado com todas as etapas do sistema alimentar, desde a relação com a terra e o uso de sementes crioulas - com uma produção sem veneno e que não explore o trabalhador; com a comercialização e o escoamento dos alimentos baseados em circuitos curtos e um consumidor consciente - até a produção e destinação de resíduos, com a utilização de embalagens biodegradáveis. Essas ações legitimam que os jovens se orientam pela sustentabilidade numa perspectiva multidimensional: ambiental, econômica, social e cultural. Na direção contra-hegemônica, as ações desenvolvidas pelos jovens se baseiam na coletividade, na solidariedade e na justiça. Foram atribuídas críticas ao sistema agroalimentar global vigente, o qual, alinhado ao capitalismo, tem como premissa a competição e o lucro.

O presente estudo contribui para o avanço nas discussões sobre alimentação sustentável sob a lente das juventudes rurais, cujos resultados podem subsidiar a construção de ações de mobilização com outros jovens, ajustadas aos interesses e peculiaridades desse potente grupo social.
